# Fluorescent protein tagging of *C. elegans* core apoptosis pathway components reveals mitochondrial localization of CED-9 Bcl-2, CED-4 Apaf1 and CED-3 Caspase in non-apoptotic and apoptotic cells

**DOI:** 10.1038/s41418-025-01567-8

**Published:** 2025-08-27

**Authors:** Eric J. Lambie, Alan Greig, Barbara Conradt

**Affiliations:** Cell and Developmental Biology, The Centre for Cell and Molecular Dynamics, Faculty of Life Sciences,, University College London, London, UK

**Keywords:** Cell biology, Genetics

## Abstract

We used CRISPR-Cas-mediated modification of the genomic loci for *C. elegans* genes *ced-9* Bcl-2, *ced-4* Apaf1 and *ced-3* Caspase to add the coding sequence for the mNeonGreen (mNG) fluorescent protein to the endogenous open reading frames. In each case, the addition of mNG caused little or no apparent alteration of gene function. We found that tagged versions of CED-9, CED-4 and CED-3 proteins colocalize with mitochondria in all cells of live mid-late stage embryos and are distributed along the entire length of mitochondria. However, CED-4 also exhibits localized puncta of ~4-fold enrichment, and these are preferentially oriented toward the nucleus. We do not observe any shift in the localization pattern of tagged CED-4 in cells that are committing to apoptosis during normal development. However, when *egl-1* BH3-only is overexpressed or *ced-9* removed by mutation, CED-4::mNG is no longer distributed along the entire length of mitochondria and instead becomes enriched in the bright puncta. Finally, localization of CED-3::mNG to mitochondria is independent of both CED-9 and CED-4. This study represents the first analysis of the distribution and sub-cellular localization of endogenous CED-9 Bcl-2, CED-4 Apaf1 and CED-3 Caspase proteins in live embryos. Our results impact the current model of apoptosis commitment in *C. elegans*.

## Introduction

Apoptotic cell death is an evolutionarily conserved mechanism for the removal of unwanted cells during animal development [1]. The identities and sequential functions of the core apoptosis pathway components were primarily determined through a series of genetic studies using *C. elegans* as a model [2–4]. Subsequent molecular characterization of the corresponding gene products revealed that the pathway comprises EGL-1 BH3-only, CED-9 BCL-2, CED-4 Apaf1 and CED-3 Caspase [5–8]. Based on data obtained via molecular, genetic, structural and cell biological studies, the following “textbook” model for the regulation of apoptosis in the somatic tissues of *C. elegans* has become popularized [9–11]: During embryogenesis, *egl-1* is transcriptionally silenced in most non-apoptotic lineages, but each of the other genes is expressed [12–15]. CED-9 BCL-2 protein is localized to the outer mitochondrial membrane, where it binds to a dimer of CED-4 Apaf1 protein. CED-3 Caspase protein is cytosolic and predominantly in the inactive pro-form. In cells fated to undergo apoptosis, *egl-1* expression is strongly upregulated. EGL-1 protein binds to CED-9, triggering a conformational change that releases CED-4. CED-4 dimers then assemble into an octameric complex (the apoptosome) that associates with the perinuclear region, recruits pro-CED-3 and promotes autocatalytic cleavage of CED-3 into its mature form. CED-3 then cleaves multiple targets, ultimately resulting in apoptotic cell death.

Although this model is appealing, some aspects are controversial, e.g., the idea that CED-9 inhibits apoptosome assembly by sequestering CED-4 on mitochondria has been questioned by multiple laboratories [12, 16–18]. Furthermore, while CED-3::GFP subcellular localization has been examined in the germ line [19], there has been no in-depth characterization in somatic cells. As a means to begin resolving some of these issues, we sought to develop genetic tools that would permit in vivo visualization of the localization of the endogenous core apoptosis pathway proteins during *C. elegans* development. To this end, we used CRISPR-Cas mediated editing of genomic loci to generate mNeonGreen (mNG)-tagged versions of CED-9, CED-4 and CED-3.

## Results

### CRISPR-Cas tagging strategy

**Fig. 1:**
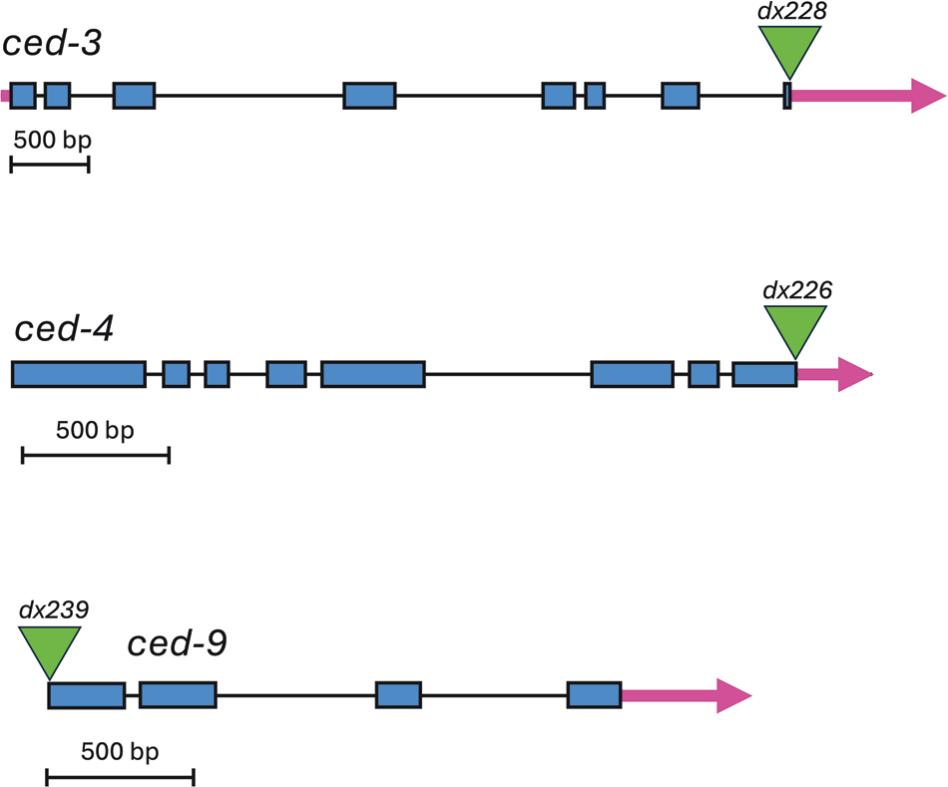
mNG insertion sites in *ced-3, ced-4* and *ced-9* loci. Gene structures drawn according to annotations on WormBase [55].

We used CRISPR-Cas9 and CRISPR-Cas12 editing to modify the genome of wild type N2 worms (see Methods) and candidate insertion alleles were sequenced to validate the structures of the edited loci. The strategy for tagging each gene is diagrammed in Fig. 1. We used published structural data [20–25] to choose tag locations that would be unlikely to interfere with protein activity, proper localization or interactions with other relevant proteins. We used mNG for tagging, because it is expected to be superior to GFP in terms of brightness and resistance to photobleaching [26].

### Functionality of tagged loci

**Fig. 2:**
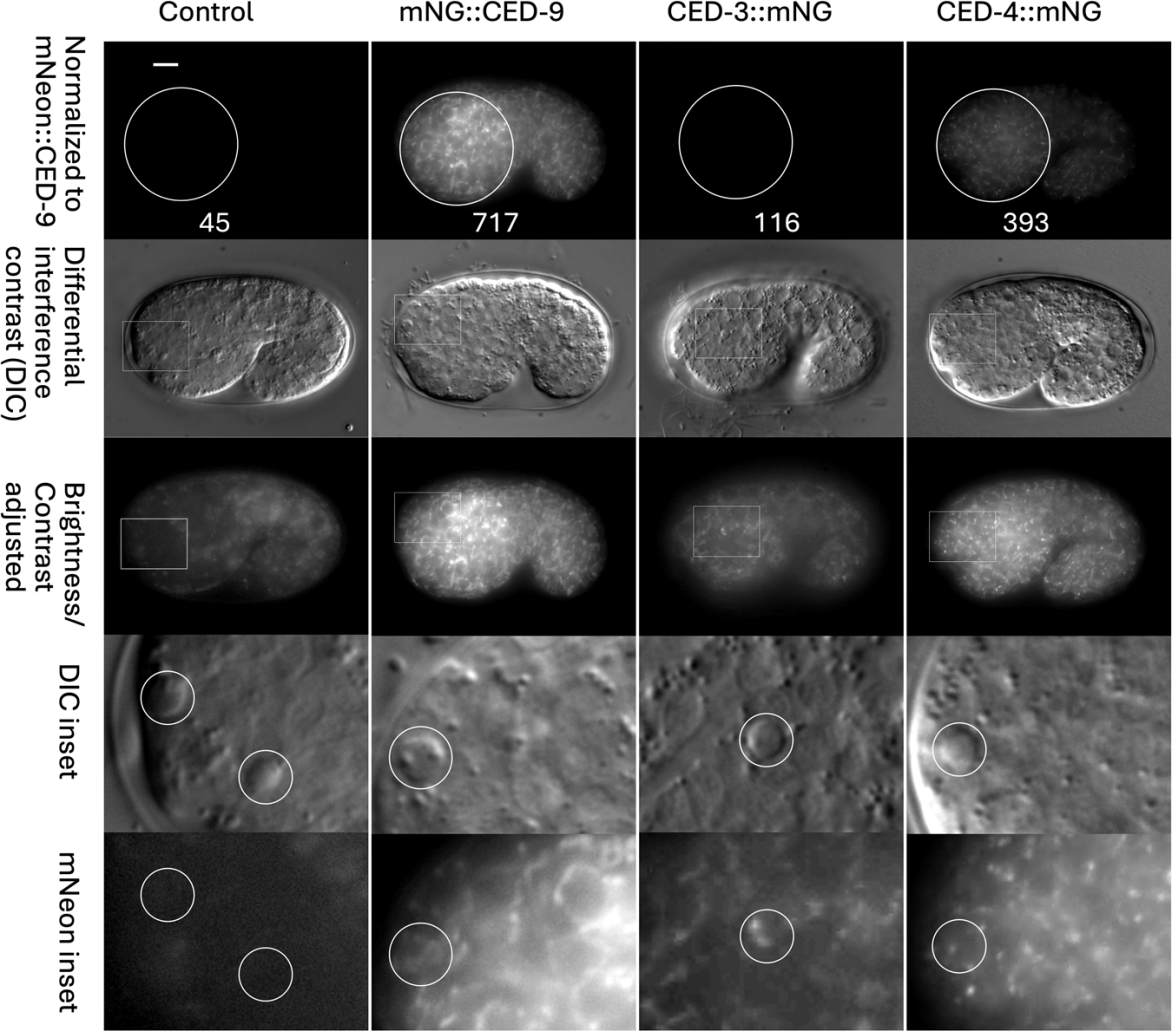
Expression and localization of mNG-tagged CED-3, CED-4 and CED-9. Images were acquired using a Zeiss AxioImager M2 as described in Methods. The top row (normalized) shows the fluorescence pattern for each strain, where each image was set to the same brightness scale in Image J. The mean pixel intensity for each embryo shown was measured within a 400 pixel diameter circular region (indicated) in order to compare relative signal strengths. Images were then converted to 8 bit for import, labeling and adjustment of brightness/contrast (row 3) in Microsoft PowerPoint^TM^. Rectangles indicate inset regions that are expanded in the bottom two rows. Circles indicate refractile apoptotic cells. All strains include *unc-29(dxIs20[rol-6(su10060); unc-3::P2A::mScarlet-I]*). The control strain lacks other modified loci. The other strains contain *ced-9(dx236; N-mNG)*, *ced-3(dx228; C-mNG)* or *ced-4(dx226; C-mNG)* as indicated. Scale bar, 10 micrometers.

We found that each of the homozygous tagged strains appears healthy and exhibits an approximately normal number of apoptotic cells in mid-embryogenesis (Fig. 2). In order to quantitatively assess the efficiency of apoptosis, we counted the number of cells in the anterior pharynx of L4 stage hermaphrodites [27]. In the case of *ced-3* and *ced-4*, a loss-of-function would be indicated by the presence of “extra cells” within this region. For example, strong loss-of-function alleles of *ced-3* have an average of 11-12 extra cells, whereas N2 has one or none [28]. In the case of *ced-9*, extra cells would indicate a gain-of-function phenotype. In all of our tagged strains, the average number of extra cells in the anterior pharynx was <0.3 (Table 1), suggesting that there is no major alteration in the activity level of the apoptosis pathway. In the case of *ced-3::mNG*, the number of extra cells is significantly elevated compared to wild type, suggesting that the insertion may slightly impair gene activity. Reduction of *ced-9* function leads to inappropriate apoptosis, which causes defective egg laying and embryonic lethality [4]. However, we did not observe any egg-laying defects (*n* > 500) or significant embryonic lethality in *mNG::ced-9* homozygotes (1/1834 for *mNG::ced-9* vs 3/2107 for N2).

**Table 1:** Assessment of inappropriate cell survival by counting cell number in the anterior pharynx.

Genotype	Average number of extra cells per pharynx	Number of animals scored	*p* value
N2	0	15	NA
*ced-3(dx228)*	0.21	14	<0.05
*ced-4(dx226)*	0.07	14	>0.05
*ced-9(dx236)*	0	12	>>0.05

*p* values were calculated using a one-tailed z-test and indicate the likelihood that the observed number of surviving cells is not greater than observed in N2 [56]. These calculations are based on the observation that Ced animals have an average of 11.4 extra cells in the anterior pharynx [8]. Sample sizes were chosen to allow detection of a significant increase in the number of surviving cells at a frequency of ≥2%. Each CRISPR-edited strain was outcrossed once to N2 prior to scoring.

### Expression and localization assayed by widefield microscopy

Each of the mNG-tagged proteins is present in all cells of the embryo, with little variation in level between cells. When the same exposure settings are used for each strain, the level of signal is highest for mNG::CED-9, followed by CED-4::mNG, and then CED-3::mNG (Fig. 2). For example, for the representative images shown in the top row of Fig. 2, the relative intensities are 9.5 (CED-9): 4.9 (CED-4): 1 (CED-3) (after subtraction of background autofluorescence). This ratio of CED-9/CED-4 (1.94) is consistent with the idea that CED-9 inhibits cell death in non-apoptotic cells by titrating CED-4. mNG::CED-9 labels tubular subcellular structures that resemble mitochondria in both non-apoptotic and apoptotic cells (Fig. 2). CED-4::mNG exhibits a subcellular pattern similar to that of mNG::CED-9, but with intermittent bright foci that produce a punctate appearance (Fig. 2). The localization pattern of CED-4::mNG is not obviously different between apoptotic and non-apoptotic cells. Although more difficult to discern due to the poor signal/background ratio, CED-3::mNG also exhibits a subcellular pattern consistent with mitochondrial localization. CED-3::mNG signal is typically brighter in apoptotic cells, as expected based on the expression pattern reported for a *ced-3* transgene [13].

**Fig. 3:**
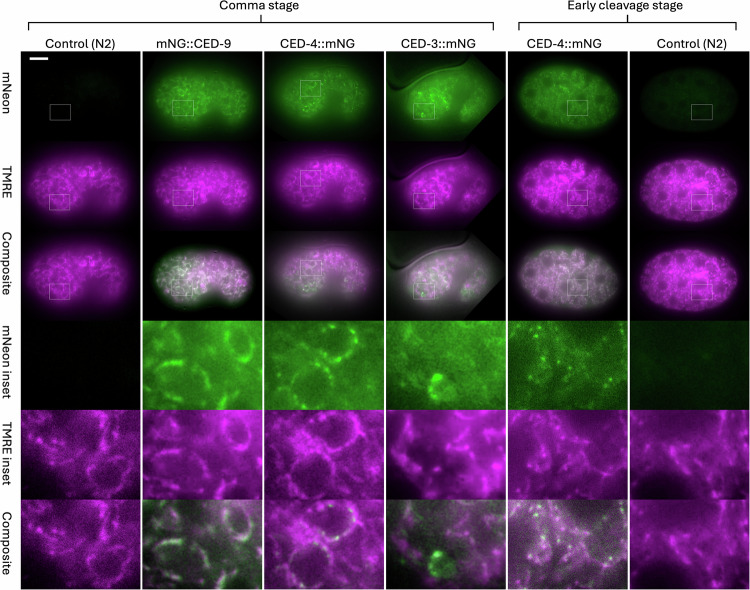
Widefield imaging of mNG tagged CED proteins and TMRE-labeled mitochondria. TMRE staining was done as described in Methods. Images were acquired using a Zeiss Elyra7 microscope in widefield mode, as described in Methods. Images were colorized and composited in ImageJ, then converted to 8 bit and imported into Microsoft PowerPoint^TM^ for labeling. Genotypes are as in Fig. 2 except that the control images are non-transgenic N2 embryos. In the top row, the brightness scale of the comma-stage N2 embryo (extreme left) was set to the same range as mNG::CED-9 in ImageJ, while the brightness scale of the early-cleavage stage N2 embryo is set to the same scale as the early-cleavage stage CED-4::mNG embryo. The brightness and contrast of other panels was adjusted as necessary to permit visibility without creating artifacts. Scale bar, 10 micrometers.

In order to directly test the idea that the subcellular localization of the mNG-tagged proteins of the apoptosis pathway overlaps with mitochondria, we stained live embryos with TMRE (Tetramethylrhodamine ethyl ester) [29], which is selectively concentrated in the mitochondrial matrix due to the inner mitochondrial membrane potential. Using widefield microscopy, we observed identical patterns in both channels when comparing mNG::CED-9 and TMRE, consistent with the known localization of CED-9 to the outer mitochondrial membrane (Fig. 3) [12, 16]. We also observed mitochondrial localization of CED-3::mNG in cells where the level of tagged protein was sufficiently high for detection above background. In the case of CED-4::mNG, we observed consistent signal that overlapped with the TMRE pattern, indicating localization to mitochondria. In cells where the focal plane is favorable for viewing, it can be seen that the bright CED-4::mNG puncta that are associated with mitochondria are consistently positioned toward the nucleus. This arrangement is characteristic of most or all of the cells in the embryo, and therefore does not correlate with apoptosis. In the large blastomeres of early cleavage stage embryos, it can be seen that the puncta are associated with mitochondria throughout the cytoplasm and not preferentially situated near the nucleus (Fig. 3).

### Super-resolution imaging of mNG-tagged proteins and TMRE-stained mitochondria

**Fig. 4:**
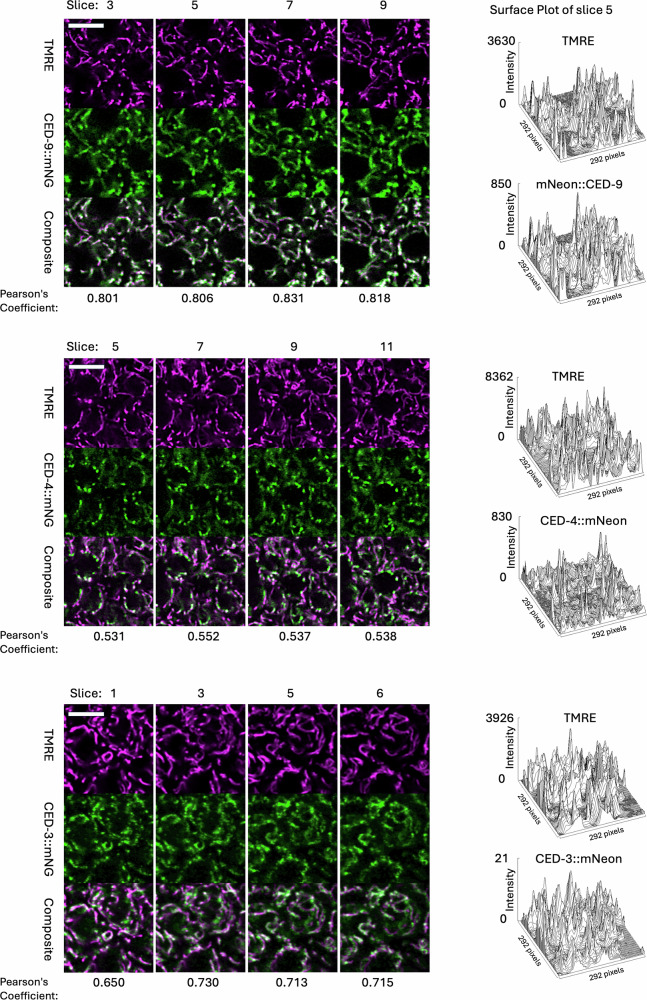
LSM 980 imaging of mNG-tagged CED proteins and TMRE-labeled mitochrondria. TMRE-stained comma-stage embryos were imaged using a Zeiss LSM 980 microscope equipped with an AiryScan 2 detector (see Methods). Genotypes are as in Fig. 2. Image stacks were acquired at 0.15 micron intervals. The slice numbers shown are indicated. Scale bar, 5 micrometers.

In order to look more precisely at the positioning of the mNG-tagged proteins relative to mitochondria, we imaged TMRE-stained live embryos using a Zeiss LSM 980 microscope equipped with an AiryScan 2 detector (Methods). In order to quantitate the degree of colocalization between the tagged CED proteins and mitochondria, we used the image J Plugin, JaCoP, to determine the Pearson’s Coefficient (PC) for mNG and TMRE signal [30]. For the image slices shown in Fig. 4, the average PC values were as follows: CED-9, 0.815; CED-3, 0.703; CED-4, 0.53. If the mNG signal is randomized using JaCoP, e.g., for slice 5, the PC values are much lower (CED-9, 0.003; CED-3, 0.005; CED-4, 0.011). The relative PC values are consistent with the qualitative comparison of the mNG patterns for CED-3 and CED-9, which appear to associate relatively uniformly with the mitochondrial surface and CED-4, which is less evenly distributed and exhibits bright puncta that have little overlap with the TMRE signal. Overall, the super-resolution imaging data support the idea that each of the tagged proteins localizes to mitochondria.

The AiryScan image data allow a quantitative estimate of the relative brightness of different objects within a given image plane [31]. Since the same acquisition settings were used for CED-4::mNG and mNG::CED-9, we can also compare their relative brightness in order to provide an approximate measure of their relative abundance. For example, the average intensity of non-zero pixels in slice five is 84.0 for mNG::CED-9 and 46.4 for CED-4::mNG. The brightness ratio (CED-9/CED-4 = 1.83) is very similar to that obtained through widefield imaging (1.94, above). Different imaging settings were used for CED-3::mNG due to its low intensity, so the brightness data are not comparable with those for CED-4 and CED-9.

The surface plots shown in Fig. 4 show the intensity distributions for mNG and TMRE in representative image slices for each strain. TMRE staining is variable between individuals, so its absolute brightness level is not informative; however, within a given slice it does provide a qualitative profile of the mitochondrial matrix. For CED-3::mNG and mNG::CED-9, the intensity profiles of TMRE and CED-9 exhibit similar contours, consistent with a relatively uniform association along the length of the mitochondrion. CED-4::mNG, as expected, exhibits peaks that are disproportionate to the TMRE signal. The peak sizes are variable, but the approximate range of intensity is ~200 to ~800, i.e., a 4-fold increase from low to high.

### Level and localization of mNG-tagged proteins within the RID lineage

**Fig. 5:**
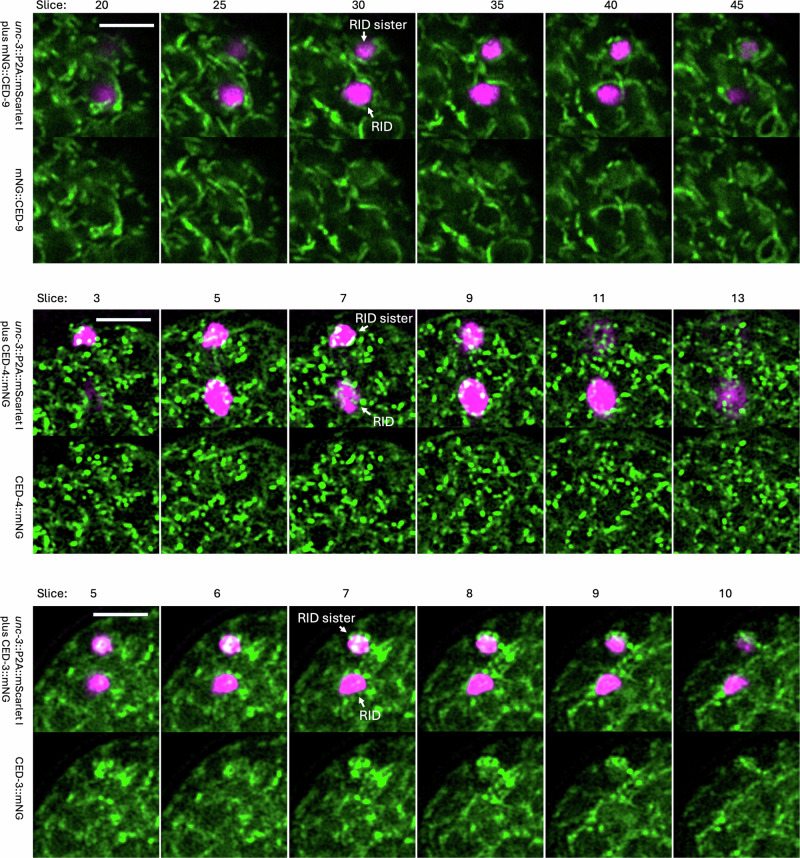
LSM 980 imaging of mNG-tagged CED proteins in RID and the sister of RID. Open arrowhead indicates RID nucleus and closed arrowhead is nucleus of RID sister. Imaging was as for Fig. 4 except that CED-3::mNG was imaged using a 25X/0.8 LD LCI Plan Apochromat objective with 0.63 micron z increments in order to reduce photobleaching. Scale bar, 5 micrometers.

In order to observe level and localization of the tagged proteins within a specific cell death lineage, we used a nuclear-localized mScarlet-I reporter that is driven by the *unc-3* promoter (Methods). *unc-3* expression begins in the mother of the RID neuron and persists in both the RID neuron and the RID sister, which undergoes apoptosis [32, 33]. The levels and localization patterns of mNG::CED-9 and CED-4::mNG are not obviously different between the RID cell and its sister (Fig. 5). However, in approximately half of the 1.5-fold embryos that we examined, CED-3::mNG signal is particularly strong in the RID sister (Fig. 5). Although we did not perform a time course, these are probably cells that have progressed farther in apoptosis. To determine whether an increase in CED-3::mNG signal is a general phenomenon observed in apoptotic cells, we analyzed 13 embryos at the comma to 1.5-fold stage and identified 54 apoptotic cells based on their refractility using Differential Interference Contrast (DIC) microscopy. We found that 22 of these apoptotic cells had mitochondria-localized CED-3::mNG signal brighter than in neighboring cells.

### CED-4::mNG localization in embryos overexpressing *egl-1*

**Fig. 6:**
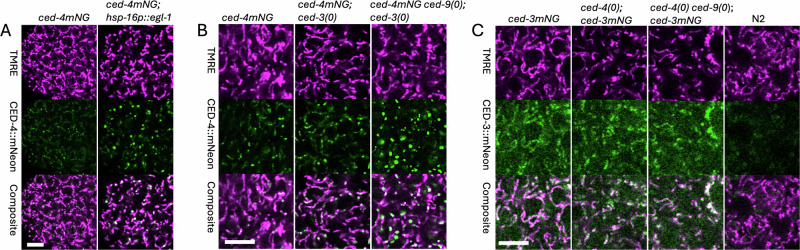
Effects of genetic manipulations on localization of mNG-tagged CED proteins. All images in this figure were acquired using the LSM980. **A** Pre-comma stage embryos derived from hermaphrodites of genotype *ced-4mNG(dx226)* and *ced-4mNG(dx226); dxEx50[hsp-16::egl-1;rol-6(su1006)]*, imaged ~2 h after a 1 h, 33 °C heatshock. Identical image acquisition and processing conditions were used for the mNG panels. **B** 1.5-fold stage embryos of genotype i) *ced-4mNG(dx226)*, ii) *ced-4mNG(dx226) ced-9(dx210); ced-3(dx211)*, and iii) *ced-4mNG(dx226)*; *ced-3(dx213)*. Identical image acquisition and processing conditions were used for *ced-4mNG(dx226)*; and *ced-4mNG(dx226) ced-9(dx210); ced-3(211)*. Different acquisition settings were used for the *ced-4mNG(dx226); ced-3(213)* strain, so the brightness of the mNG panel was manually adjusted to match that in the *ced-4mNG(dx226)* panel and reveal the weak mNG signal extending along the mitochondrial length. **C** 1.5-fold stage embryos of genotype i) *unc-29(dxIs20[rol-6(su1006); unc-3::P2A::mScarlet-I]*)*; ced-3mNG(dx228)*, ii) *ced-4(n1162); ced-3mNG(dx228)*, iii) *unc-29(dxIs20[rol-6(su1006); unc-3::P2A::mScarlet-I]*); *ced-4(n1162) ced-9(dx215); ced-3mNG(dx228)* and iv) wild type N2. Identical image acquisition and processing conditions were used for all mNG panels.

Although we did not observe any obvious relocalization of CED-4::mNG in cells that commit to apoptosis during wild type development (Fig. 5), we wondered whether this could be due to incomplete release of CED-4::mNG from CED-9. Therefore, we tested whether transgene-mediated, heat-shock induced overexpression of *egl-1* BH3-only would alter the localization pattern of CED-4::mNG. Indeed, we found that some embryos exhibited a reduction in localization of CED-4::mNG along the entire mitochondrial length concomitant with an increase in signal within mitochondria-associated puncta (Fig. 6A). This appears to be due to relocalization, because the average intensity of mNG across the embryo is only marginally different than control embryos; e.g., the average mNG signal in the control embryo in Fig. 6A is 570 brightness units compared to 605 for the heat-shock *egl-1* embryo.

### CED-4::mNG localization in the absence of CED-9

As a more direct test of whether complete release of CED-4::mNG from CED-9 would cause a shift in localization, we sought to inactivate *ced-9* genetically. Since the *ced-4* and *ced-9* loci are very tightly linked on chromosome III, we used CRISPR-Cas editing to delete *ced-9* on a *ced-4::mNG* chromosome (see Methods). In order to rescue the embryonic lethal phenotype of *ced-9(0)*, we simultaneously deleted *ced-3* on chromosome IV. In the resulting strain, *ced-4(dx226mNG) ced-9(dx210)* III; *ced-3(dx211)* IV, we observed that the low level of CED-4::mNG all along mitochondria is no longer present. However, the mitochondria-associated puncta are larger and brighter (Fig. 6B), and the average level of mNG signal across the embryo is more than twice as high (709 vs. 281; average of three different embryos each). The peak values are also higher as expected (38,953 vs 11,203; average of three different embryos each) since the puncta are clearly brighter. The shift in localization pattern of CED-4::mNG is not due to the *ced-3(dx211)* mutation, because a derivative strain of genotype *ced-4(mNG)* III; *ced-3(dx211)* IV has the same distribution of CED-4::mNG as in wild type (Fig. 6B).

### CED-3::mNG level and localization in the absence of CED-4 and/or CED-9

Published reports that CED-3 physically interacts with CED-4 and CED-9 predict that mitochondrial localization of CED-3 should be dependent upon CED-4 and/or CED-9. To determine whether CED-3::mNG level and/or localization is dependent on CED-4, we examined the effect of the null allele, *ced-4(n1162)* [6]. We found that mitochondrial localization of CED-3::mNG during embryogenesis was not obviously affected by the absence of CED-4 (Fig. 6C). To test for dependency on CED-9, we generated a deletion allele, *ced-9(dx215)* using a strain of genotype *ced-4(n1162)*. In this strain background (*ced-4(n1162) ced-9(dx215)* III), the localization and abundance of CED-3::mNG appear to be unchanged (Fig. 6C). With the imaging conditions used in these experiments (35 times faster scan speed compared to CED-3::mNG data shown in Fig. 4), it is evident that there is a significant amount of above-background non-mitochondrial signal in the mNG channel. This suggests that CED-3::mNG is not exclusively localized to mitochondria.

## Discussion

We find that the tagged versions of CED-9 Bcl-2, CED-4 Apaf-1 and CED-3 Caspase proteins are all ubiquitously present in *C. elegans* embryos and localize predominantly to mitochondria. We acknowledge the possibility that the insertion of mNG coding sequences could affect abundance or subcellular localization of the tagged proteins. Furthermore, addition of mNG to the tagged proteins could potentially alter their stability (either increase or decrease) or otherwise affect their intrinsic functions. However, we do not observe any major loss- (*ced-9*, *ced-4*, *ced-3*) or gain-of-function (*ced-9*) phenotypes in the modified strains. Therefore, any such effects are either of low magnitude or have minimal impacts on the normal functions of these genes.

The subcellular localization of CED-9 and CED-4 to mitochondria in non-apoptotic cells during embryogenesis is consistent with previous reports [12, 16]. The presence of CED-3 on mitochondria was not anticipated. However, the subcellular pattern of germline-expressed CED-3::GFP reported by Chen et al. [19] does bear a strong resemblance to mitochondria. The mechanism through which CED-3 is targeted to mitochondria is not known, but there are some hints as to how this could potentially be accomplished. First, CED-4 has been shown to bind to the 3’ UTR of *ced-3* mRNA [34], and this could conceivably promote localized translation of CED-3 on the mitochondrial surface. (And CED-4 and CED-3 proteins have been shown to interact.) However, we found that neither the translation of *ced-3* mRNA nor the mitochondrial localization of CED-3::mNG protein were dependent upon CED-4. Second, CED-3 could be recruited to the mitochondrial surface by CED-9, since these proteins have been shown to associate in vivo when overexpressed in mammalian cells [35, 36]. However, we observed no obvious effect on CED-3::mNG localization or abundance in embryos that lack CED-9 protein. A third possibility is suggested by our observation here that CED-3 has both a weak N-terminal mitochondrial targeting sequence and multiple strong internal mitochondrial targeting sequences (Supplementary Fig. S1); together, these would be predicted to promote binding of CED-3 to TOM-20 and TOM-70 on the outer mitochondrial membrane [37].

While CED-3::mNG is clearly localized to mitochondria, we also observe above-background signal across the rest of the cell. However, we do not know what fraction of total CED-3::mNG protein is localized to mitochondria. Furthermore, it remains unknown what fraction of the CED-3::mNG that we observe represents active Caspase, because the C-terminal mNG tag does not allow us to distinguish between pro-CED-3 and processed CED-3. Since the candidate substrates for CED-3 are localized in multiple cellular compartments, we consider it unlikely that CED-3 caspase activity is confined to the mitochondrial surface.

We observe that CED-4::mNG is unevenly distributed on the mitochondrial surface, with bright regions of ~4-fold higher signal level. These bright puncta have typically been interpreted as characteristic of perinuclear localization [12, 16, 17, 38]. However, CED-4::mNG puncta are present in early cleavage stage embryos and are clearly not perinuclear at this stage. We do observe that the CED-4::mNG puncta are oriented toward the nucleus in mid-late stage embryos, but they continue to be closely associated with mitochondria.

It has been proposed that the localization of CED-4 shifts from mitochondrial to perinuclear (i.e., puncta) when cells undergo apoptosis [12, 16]. We do not observe any obvious change in the pattern of CED-4 localization in cells undergoing apoptosis during wild type development. However, either deletion of *ced-9* or overexpression of *egl-1* causes a shift in the distribution of CED-4::mNG, with signal becoming confined to the bright puncta, which remain associated with mitochondria.

**Fig. 7:**
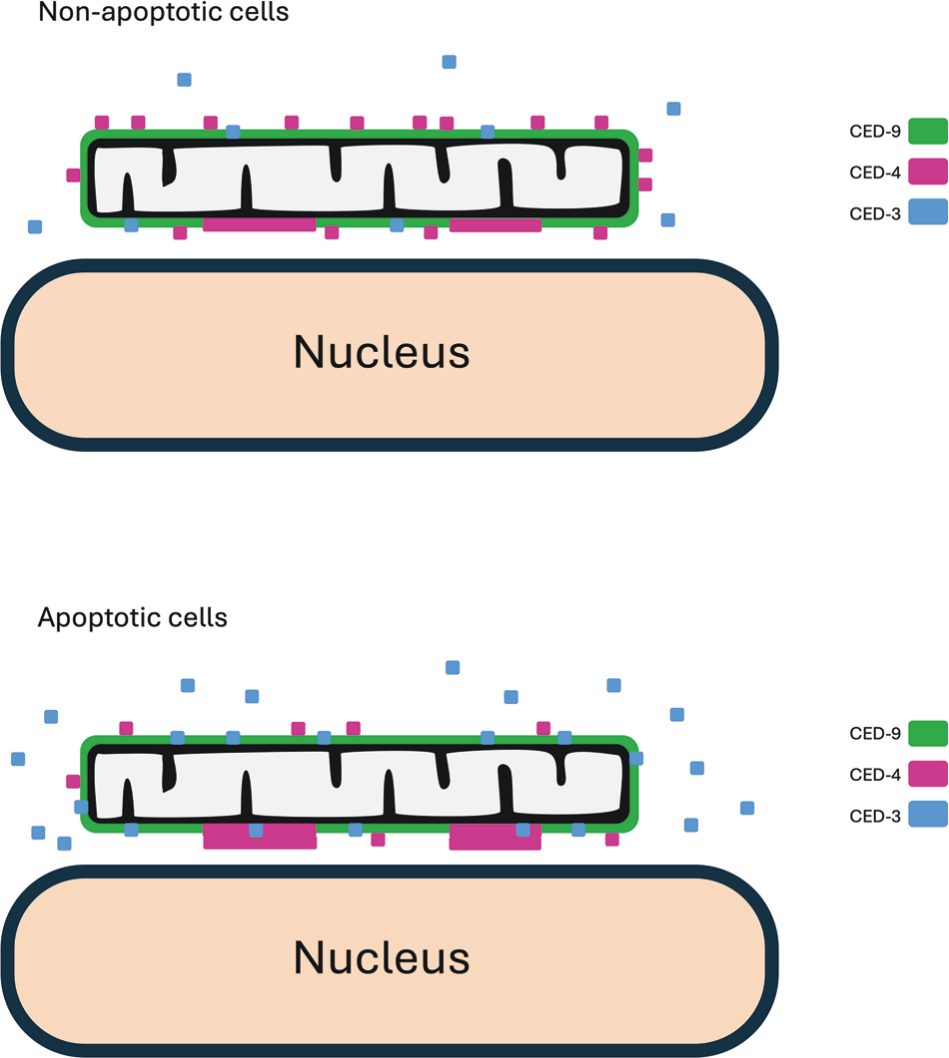
Schematic of subcellular localization of CED proteins in non-apoptotic and apopotic cells in the embryo. See text for proposed mechanistic details.

Based on our analyses, we propose the following model for the sequence of events leading to the induction of apoptosis (Fig. 7). In non-apoptotic, somatic cells of the embryo, CED-9 localizes to the outer mitochondrial membrane, where it is present in approximately 2-fold molar excess to CED-4 overall. CED-9 binds to dimers of CED-4, and these complexes are distributed along the length of the mitochondrion. A fraction of CED-4 protein undergoes oligomerization into incomplete apoptosomes (hexamers or heptamers [24]), which appear as bright puncta that are associated with mitochondria. CED-9 prevents assembly of the complete apoptosome, but is not required for the mitochondrial association of the CED-4 puncta. pro-CED-3 binds to TOM-20 and TOM-70, but is not recruited to the immature apoptosomes. In cells that undergo apoptosis, the transcription of *egl-1* [15, 39, 40] is induced at high levels. EGL-1 protein rapidly accumulates and binds to CED-9. This triggers a conformational change in CED-9 that permits completion of apoptosome assembly (presumably by releasing CED-4 dimers) and processing of pro-CED-3. It has been suggested that processed CED-3 remains associated with the apoptosome to form a holoenzyme. However, we do not observe enrichment of CED-3::mNG at CED-4 puncta in cells undergoing apoptosis. Possibly, the mNG tag interferes with this association and this is why we observe a weak loss-of-function phenotype in the CRISPR modified strain. *ced-3* transcription is also increased in cells that commit to apoptosis and this leads to production of additional pro-CED-3 [13]. Consequently, a positive feedback loop is initiated, with CED-3 caspase promoting further CED-4/apoptosome activity by inactivation of CED-9 through proteolytic cleavage [41].

The CED-4::mNG puncta that we observe are superficially similar to the Apaf-1::GFP foci reported by Borgeaud et al. [42]. These authors found that GFP-tagged Apaf1 undergoes aggregation into easily visualized cytoplasmic puncta in cultured mammalian cells that were induced to undergo apoptosis. However, these aggregates were not preferentially associated with mitochondria, and their formation was dependent on caspase activity.

It remains to be determined how the CED-4 puncta are physically associated with mitochondria. Our results suggest that sequestration of CED-4 along mitochondria by CED-9 prevents CED-4 from undergoing oligomerization and that aggregated CED-4 may be resistant to turnover. These are consistent with the known anti-apoptotic activities of CED-9. The pro-apoptotic function of CED-9 is less well understood [16, 28, 43], but our observations suggest that CED-9 could facilitate association between CED-4 and CED-3 on the mitochondrial surface.

## Methods

### *C. elegans* culture

Standard methods were used for *C. elegans* culture and genetic manipulations [44], except bacterial strain AMA1004 was used as a food source [45].

### CRISPR-Cas-mediated editing

Wild type *C. elegans* N2 (Brenner 1974) was used as the starting strain for CRISPR modifications and for outcrossing edited strains. CRISPR editing was done essentially as described by Paix et al. [46], but in some cases ssDNA repair templates generated by asymmetric PCR were used on the recommendation of Eroglu et al. [47]. In some experiments, *unc-29* was used as a Co-CRISPR marker instead of *dpy-10*. F1 animals with successful edits were identified by screening for mNG fluorescence then verified by PCR and sequencing. Cas9, Cas12a and TracrRNA for use in CRISPR editing were obtained from IDT (Coralville, Iowa). Cas9 and Cas12a crRNA sequences were identified using CRISPOR (http://crispor.gi.ucsc.edu/crispor.py) [48] and/or the IDT design tool. Some crRNAs were purchased from Sigma.

### TMRE staining

TMRE was from Molecular Probes (Thermo Fisher Scientific). TMRE staining was done by culturing worms overnight on seeded NGM plates containing 100 nM TMRE and then imaging embryos mounted on 3% agarose pads.

### Microscopy

Routine widefield imaging was done using a Zeiss AxioImager M2. Standard DIC optics were used, and imaging was done using an EC Plan-Neofluar 100×/1.3 oil objective. For mNG imaging the same objective was used, but with a Colibri 7 LED light source 450–488 nm in combination with filter set 90. Images were acquired with a Photometrics Prime BSI sCMOS camera (USA) using ZEN Blue 2.6 software. For widefield imaging of TMRE plus mNG, we used a Zeiss Elyra7 microscope with a Plan-Apochromat 63×/1.4 oil objective. We used simultaneous excitation at 488 nm (mNG plus TMRE) and 561 nm (TMRE only), and a dual camera bandpass filter system to separately capture emission from 495 to 550 nm (mNG only) and >570 nm (TMRE plus a minor fraction of mNG).

For super resolution imaging, we used a Zeiss LSM 980 plus AiryScan 2 detector system. Imaging of mNG was done using 488 nm excitation and >509 nm emission. Imaging of mScarlet-I and TMRE were done separately from mNG using 552 nm excitation and >578 nm emission. Acquisition parameters were adjusted in ZEN Blue 3.3 as necessary to optimize signal/noise ratio without excessive photobleaching. Standard autofilter settings were used for AiryScan processing.

At least ten embryos were imaged for each genotype and experimental condition reported. Representative examples are shown in figures. Each strain imaged represents a single biological replicate. Subcellular localization of tagged CED proteins was assessed in a minimum of three experiments (widefield AxioImager, widefield Elyra, Confocal LSM 980). Each CED protein localization in reponse to genetic manipulation was assessed visually using the AxioImager, then documented on the LSM 980.

### Transgene construction

Codon optimization and intron placement for mNG and mScarlet-I were determined using the *C. elegans* codon adapter tool (https://worm.mpi-cbg.de/codons/cgi-bin/optimize.py) [49]. Oligonucleotides for PCR were from Merck Life Science (UK). Q5, LongAmp DNA polymerase and 2X HiFi cloning mix were from NEB (UK). Electrocompetent DH10B cells were from Thermo Fisher Scientific (UK).

The RID lineage reporter, *unc-29(dxIs20[unc-3-P2A-NLSmScarlet; pRF4)* I, was generated by in vivo recombination [50] followed by CRISPR-Cas-mediated integration as follows. First, plasmid pEL297 was generated, carrying codon-optimized nuclear localized mScarlet-I [51] followed by 2100 bp of sequence immediately downstream of the *unc-3* coding region. pEL297 was used as a PCR template to amplify a 3058 bp product that begins with 25 bp of homology at the AflII site at the 3’ end of *unc-3* coding sequence, followed by the coding region for a P2A self-cleaving peptide sequence [52], NLSmScarlet-I coding sequences, and 2 kb downstream of *unc-3*. A mixture was then prepared containing 20 μg/ml of this PCR fragment, 20 μg/ml of *unc-3* genomic fosmid WRM0618C_G12 that had been cleaved with AflII, and 125 μg/ml of coinjection marker pRF4(*rol-6(su1006)*) [53]. This mixture was then injected into wild type N2 worms and progeny screened to obtain a line of healthy mScarlet(+) Rol animals carrying an extrachromosomal array. In order to integrate the array, we used a strategy similar to that described by Yoshina et al. [54]: mScarlet(+) Rol animals were injected with a mix containing Alt-R^TM^ Cas9 (1.4 mg/ml), plus an sgRNA that targets the *unc-29* genomic locus (UGACACCUCGUAAUUUCCAU) (5.4 microMolar), an sgRNA that targets the colE1 origin (GCUACCAACUCUUUUUCCGA) (5.4 microMolar) and an sgRNA that targets a sequence in the 3’ flank of *rol-6* (AAGAAAGUUCUUAACAUCCA) (5.4 microMolar). Prospective integration-carrrying Unc-29 F1s were identified by screening for tetramisole resistance [44] and mScarlet expression. These were then singled and their progeny screened for high frequency transmission of mScarlet(+) and Rol in combination with healthy growth and low frequency of embryonic lethality. Candidate integrants were outcrossed with N2 males to verify linkage to *unc-29*.

The extrachromosomal array, *dxEx50[hsp-16p::egl-1;rol-6(su1006)]*, was made by injecting a mixture of 100 micrograms/ml pRF4 [53] plus approximately 15 micrograms/ml of *hsp-16.1p::egl-1* and 15 micrograms/ml of *hsp-16.2p::egl-1*.

## Supplementary information

Lambie et al_Supplemental Information and Figure S1

## Data Availability

All data and material used in this manuscript are available and can be requested from the corresponding authors.

## References

[1] Kerr JF, Wyllie AH, Currie AR. Apoptosis: a basic biological phenomenon with wide-ranging implications in tissue kinetics. Br J Cancer. 1972;26:239–57. 4561027 10.1038/bjc.1972.33 PMC2008650

[2] Trent C, Tsung N, Horvitz HR. Egg-laying defective mutants of the nematode Caenorhabditis elegans. Genetics. 1983;104:619–47. 11813735 10.1093/genetics/104.4.619 PMC1202130

[3] Ellis HM, Horvitz HR. Genetic control of programmed cell death in the nematode C. elegans. Cell. 1986;44:817–29. 3955651 10.1016/0092-8674(86)90004-8

[4] Hengartner MO, Ellis RE, Horvitz HR. Caenorhabditis elegans gene ced-9 protects cells from programmed cell death. Nature. 1992;356:494–9. 1560823 10.1038/356494a0

[5] Yuan J, Shaham S, Ledoux S, Ellis HM, Horvitz HR. The C. elegans cell death gene ced-3 encodes a protein similar to mammalian interleukin-1 beta-converting enzyme. Cell. 1993;75:641–52. 8242740 10.1016/0092-8674(93)90485-9

[6] Yuan J, Horvitz HR. The Caenorhabditis elegans cell death gene ced-4 encodes a novel protein and is expressed during the period of extensive programmed cell death. Development. 1992;116:309–20. 1286611 10.1242/dev.116.2.309

[7] Hengartner MO, Horvitz HR. C. elegans cell survival gene ced-9 encodes a functional homolog of the mammalian proto-oncogene bcl-2. Cell. 1994;76:665–76. 7907274 10.1016/0092-8674(94)90506-1

[8] Conradt B, Horvitz HR. The C. elegans protein EGL-1 is required for programmed cell death and interacts with the Bcl-2-like protein CED-9. Cell. 1998;93:519–29. 9604928 10.1016/s0092-8674(00)81182-4

[9] Conradt B, Wu Y-C, Xue D. Programmed cell death during Caenorhabditis elegans development. Genetics. 2016;203:1533–62. 27516615 10.1534/genetics.115.186247 PMC4981262

[10] Conradt B. Genetic control of programmed cell death during animal development. Annu Rev Genet. 2009;43:493–523. 19886811 10.1146/annurev.genet.42.110807.091533 PMC2806233

[11] Lettre G, Hengartner MO. Developmental apoptosis in C. elegans: a complex CEDnario. Nat Rev Mol Cell Biol. 2006;7:97–108. 16493416 10.1038/nrm1836

[12] Chen F, Hersh BM, Conradt B, Zhou Z, Riemer D, Gruenbaum Y, et al. Translocation of C. elegans CED-4 to nuclear membranes during programmed cell death. Science. 2000;287:1485–9. 10688797 10.1126/science.287.5457.1485

[13] Maurer CW, Chiorazzi M, Shaham S. Timing of the onset of a developmental cell death is controlled by transcriptional induction of the C. elegans ced-3 caspase-encoding gene. Development. 2007;134:1357–68. 17329362 10.1242/dev.02818

[14] Chakraborty S, Lambie EJ, Bindu S, Mikeladze-Dvali T, Conradt B. Engulfment pathways promote programmed cell death by enhancing the unequal segregation of apoptotic potential. Nat Commun. 2015;6:10126. 26657541 10.1038/ncomms10126 PMC4682117

[15] Conradt B, Horvitz HR. The TRA-1A sex determination protein of C. elegans regulates sexually dimorphic cell deaths by repressing the egl-1 cell death activator gene. Cell. 1999;98:317–27. 10458607 10.1016/s0092-8674(00)81961-3

[16] Tucker N, Reddien P, Hersh B, Lee D, Liu MHX, Horvitz HR. The pro-apoptotic function of the C. elegans BCL-2 homolog CED-9 requires interaction with the APAF-1 homolog CED-4. Sci Adv. 2024;10:eadn0325. 39383227 10.1126/sciadv.adn0325 PMC11817491

[17] Pourkarimi E, Greiss S, Gartner A. Evidence that CED-9/Bcl2 and CED-4/Apaf-1 localization is not consistent with the current model for C. elegans apoptosis induction. Cell Death Differ. 2012;19:406–15. 21886181 10.1038/cdd.2011.104 PMC3278724

[18] Tan FJ, Fire AZ, Hill RB. Regulation of apoptosis by C. elegans CED-9 in the absence of the C-terminal transmembrane domain. Cell Death Differ. 2007;14:1925–35. 17703231 10.1038/sj.cdd.4402215 PMC3047747

[19] Chen X, Wang Y, Chen Y-Z, Harry BL, Nakagawa A, Lee E-S, et al. Regulation of CED-3 caspase localization and activation by C. elegans nuclear-membrane protein NPP-14. Nat Struct Mol Biol. 2016;23:958–64. 27723735 10.1038/nsmb.3308 PMC5484413

[20] Yan N, Gu L, Kokel D, Chai J, Li W, Han A, et al. Structural, biochemical, and functional analyses of CED-9 recognition by the proapoptotic proteins EGL-1 and CED-4. Mol Cell. 2004;15:999–1006. 15383288 10.1016/j.molcel.2004.08.022

[21] Yan N, Xu Y, Shi Y. 2:1 Stoichiometry of the CED-4-CED-9 complex and the tetrameric CED-4: insights into the regulation of CED-3 activation. Cell Cycle. 2006;5:31–34. 16294007 10.4161/cc.5.1.2263

[22] Qi S, Pang Y, Hu Q, Liu Q, Li H, Zhou Y, et al. Crystal structure of the Caenorhabditis elegans apoptosome reveals an octameric assembly of CED-4. Cell. 2010;141:446–57. 20434985 10.1016/j.cell.2010.03.017

[23] Huang W, Jiang T, Choi W, Qi S, Pang Y, Hu Q, et al. Mechanistic insights into CED-4-mediated activation of CED-3. Genes Dev. 2013;27:2039–48. 24065769 10.1101/gad.224428.113 PMC3792479

[24] Li Y, Tian L, Zhang Y, Shi Y. Structural insights into CED-3 activation. Life Sci Alliance. 2023;6:e202302056. 37402593 10.26508/lsa.202302056 PMC10320015

[25] Yan N, Chai J, Lee ES, Gu L, Liu Q, He J, et al. Structure of the CED-4-CED-9 complex provides insights into programmed cell death in Caenorhabditis elegans. Nature. 2005;437:831–7. 16208361 10.1038/nature04002

[26] Shaner NC, Lambert GG, Chammas A, Ni Y, Cranfill PJ, Baird MA, et al. A bright monomeric green fluorescent protein derived from Branchiostoma lanceolatum. Nat Methods. 2013;10:407–9. 23524392 10.1038/nmeth.2413 PMC3811051

[27] Schwartz HT. A protocol describing pharynx counts and a review of other assays of apoptotic cell death in the nematode worm Caenorhabditis elegans. Nat Protoc. 2007;2:705–14. 17406633 10.1038/nprot.2007.93

[28] Shaham S, Horvitz HR. Developing Caenorhabditis elegans neurons may contain both cell-death protective and killer activities. Genes Dev. 1996;10:578–91. 8598288 10.1101/gad.10.5.578

[29] Ehrenberg B, Montana V, Wei MD, Wuskell JP, Loew LM. Membrane potential can be determined in individual cells from the nernstian distribution of cationic dyes. Biophys J. 1988;53:785–94. 3390520 10.1016/S0006-3495(88)83158-8 PMC1330255

[30] Bolte S, Cordelières FP. A guided tour into subcellular colocalization analysis in light microscopy. J Microsc. 2006;224:213–32. 17210054 10.1111/j.1365-2818.2006.01706.x

[31] Huff J. The Airyscan detector from ZEISS: confocal imaging with improved signal-to-noise ratio and super-resolution. Nat Methods. 2015;12:i–ii.

[32] Wang J, Chitturi J, Ge Q, Laskova V, Wang W, Li X, et al. The C. elegans COE transcription factor UNC-3 activates lineage-specific apoptosis and affects neurite growth in the RID lineage. Development. 2015;142:1447–57. 25790851 10.1242/dev.119479

[33] Sulston JE, Schierenberg E, White JG, Thomson JN. The embryonic cell lineage of the nematode Caenorhabditis elegans. Dev Biol. 1983;100:64–119. 6684600 10.1016/0012-1606(83)90201-4

[34] Wang M-X, Itoh M, Li S, Hida Y, Ohta K, Hayakawa M, et al. CED-4 is an mRNA-binding protein that delivers ced-3 mRNA to ribosomes. Biochem Biophys Res Commun. 2016;470:48–53. 26740177 10.1016/j.bbrc.2015.12.102

[35] Chaudhary D, O’Rourke K, Chinnaiyan AM, Dixit VM. The death inhibitory molecules CED-9 and CED-4L use a common mechanism to inhibit the CED-3 death protease. J Biol Chem. 1998;273:17708–12. 9651369 10.1074/jbc.273.28.17708

[36] Chinnaiyan AM, O’Rourke K, Lane BR, Dixit VM. Interaction of CED-4 with CED-3 and CED-9: a molecular framework for cell death. Science. 1997;275:1122–6. 9027312 10.1126/science.275.5303.1122

[37] Jung F, Rödl S, Herrmann JM, Mühlhaus T. Analysis and prediction of internal mitochondrial targeting signals. Methods Enzymol. 2024;706:263–83. 39455219 10.1016/bs.mie.2024.07.038

[38] Tzur YB, Margalit A, Melamed-Book N, Gruenbaum Y. Matefin/SUN-1 is a nuclear envelope receptor for CED-4 during Caenorhabditis elegans apoptosis. Proc Natl Acad Sci USA. 2006;103:13397–402. 16938876 10.1073/pnas.0604224103 PMC1569175

[39] Thellmann M, Hatzold J, Conradt B. The Snail-like CES-1 protein of C. elegans can block the expression of the BH3-only cell-death activator gene egl-1 by antagonizing the function of bHLH proteins. Development. 2003;130:4057–71. 12874127 10.1242/dev.00597

[40] Sherrard R, Luehr S, Holzkamp H, McJunkin K, Memar N, Conradt B. miRNAs cooperate in apoptosis regulation during C. elegansdevelopment. Genes Dev. 2017;31:209–22. 28167500 10.1101/gad.288555.116 PMC5322734

[41] Xue D, Horvitz HR. Caenorhabditis elegans CED-9 protein is a bifunctional cell-death inhibitor. Nature. 1997;390:305–8. 9384385 10.1038/36889

[42] Borgeaud AC, Ganeva I, Klein C, Stooss A, Ross-Kaschitza D, Wu L, et al. Large transient assemblies of Apaf1 constitute the apoptosome in cells. bioRxiv. 2024. 10.1101/2024.07.01.600688. 10.1038/s41467-025-64478-9 PMC12552632 41136486

[43] Jagasia R, Grote P, Westermann B, Conradt B. DRP-1-mediated mitochondrial fragmentation during EGL-1-induced cell death in C. elegans. Nature. 2005;433:754–60. 15716954 10.1038/nature03316

[44] Brenner S. The genetics of Caenorhabditis elegans. Genetics. 1974;77:71–94. 4366476 10.1093/genetics/77.1.71 PMC1213120

[45] Casadaban MJ, Martinez-Arias A, Shapira SK, Chou J. Beta-galactosidase gene fusions for analyzing gene expression in escherichia coli and yeast. Methods Enzymol. 1983;100:293–308. 6312261 10.1016/0076-6879(83)00063-4

[46] Paix A, Folkmann A, Rasoloson D, Seydoux G. High efficiency, homology-directed genome editing in Caenorhabditis elegans using CRISPR-Cas9 ribonucleoprotein complexes. Genetics. 2015;201:47–54. 26187122 10.1534/genetics.115.179382 PMC4566275

[47] Eroglu M, Yu B, Derry WB. Efficient CRISPR/Cas9 mediated large insertions using long single-stranded oligonucleotide donors in C. elegans. FEBS J. 2023;290:4429–39. 10.1111/febs.16876 37254814

[48] Concordet J-P, Haeussler M. CRISPOR: intuitive guide selection for CRISPR/Cas9 genome editing experiments and screens. Nucleic Acids Res. 2018;46:W242–W245. 29762716 10.1093/nar/gky354 PMC6030908

[49] Redemann S, Schloissnig S, Ernst S, Pozniakowsky A, Ayloo S, Hyman AA, et al. Codon adaptation-based control of protein expression in C. elegans. Nat Methods. 2011;8:250–2. 21278743 10.1038/nmeth.1565

[50] Kemp BJ, Hatzold J, Sternick LA, Cornman-Homonoff J, Whitaker JM, Tieu PJ, et al. In vivo construction of recombinant molecules within the Caenorhabditis elegans germ line using short regions of terminal homology. Nucleic Acids Res. 2007;35:e133–e133. 17933760 10.1093/nar/gkm857 PMC2095804

[51] Bindels DS, Haarbosch L, van Weeren L, Postma M, Wiese KE, Mastop M, et al. mScarlet: a bright monomeric red fluorescent protein for cellular imaging. Nat Methods. 2017;14:53–56. 27869816 10.1038/nmeth.4074

[52] Hu T, Fu Q, Chen P, Zhang K, Guo D. Generation of a stable mammalian cell line for simultaneous expression of multiple genes by using 2A peptide-based lentiviral vector. Biotechnol Lett. 2009;31:353–9. 19034387 10.1007/s10529-008-9882-3

[53] Mello CC, Kramer JM, Stinchcomb D, Ambros V. Efficient gene transfer in C.elegans: extrachromosomal maintenance and integration of transforming sequences. EMBO J. 1991;10:3959–70. 1935914 10.1002/j.1460-2075.1991.tb04966.x PMC453137

[54] Yoshina S, Suehiro Y, Kage-Nakadai E, Mitani S. Locus-specific integration of extrachromosomal transgenes in C. elegans with the CRISPR/Cas9 system. Biochem Biophys Rep. 2016;5:70–76. 28955808 10.1016/j.bbrep.2015.11.017 PMC5600330

[55] Sternberg PW, Van Auken K, Wang Q, Wright A, Yook K, Zarowiecki M, et al. WormBase 2024: status and transitioning to Alliance infrastructure. Genetics. 2024;227. 10.1093/genetics/iyae050. 10.1093/genetics/iyae050 PMC11075546 38573366

[56] Moore DS, McCabe GP. Introduction to the practice of statistics. 3rd ed. New York, NY: W.H. Freeman; 1998.

