# Supplementary material for: Fluorescent protein tagging of C. elegans core apoptosis pathway components reveals mitochondrial localization of CED-9 Bcl-2, CED-4 Apaf1 and CED-3 Caspase in non-apoptotic and apoptotic cells
Source: Cell Death Differ. 2025 Aug 27;33(1):15–24. doi: 10.1038/s41418-025-01567-8 (PMC12811295; doi:10.1038/s41418-025-01567-8)
Supplement: Supplementary file 1 — Lambie et al_Supplemental Information and Figure S1 [file 41418_2025_1567_MOESM1_ESM.docx]

**Supplemental information**

**Generation and structure of CRISPR-tagged loci**

***ced-3(dx228)***

The Cas12a crRNA sequence, GCCGGAAGCACGAAACUCUGC, was used to direct cleavage near the 3' end of *ced-3* coding sequences.

The sequence of the repair template, with homology arms indicated by underlining:

aaagttctacttctggccggaagcacgaaactctgccgtcGGTTCTGGAGCCGGGGCTTCAGGCGGTATGCCAGGATCTAAGGGAGAAGAGGATAACATGGCTTCCCTTCCAGCTACTCACGAACTTCATATTTTCGGATCTATCAACGGAGTTGATTTCGATATGGTTGGACAGgtaagtttaaacatatatatactaactaaccctgattatttaaattttcagGGAACTGGAAATCCAAACGATGGATACGAAGAGCTTAACCTTAAGTCTACCAAGGGAGATCTTCAATTCTCTCCATGGATCCTTGTCCCACACATCGGATACGGATTCCACCAATACCTCCCATACCCAGACGGAATGTCCCCATTCCAAGCTGCTATGGTTGATGGATCTGGATACCAAGTTCACCGCACTATGCAATTCGAGGATGGAGCTTCCCTTACCGTTAACTACCGCTACACTTACGAAGGATCTCACATCAAGGGAGAAGCTCAAGTTAAGGGAACTGGATTCCCAGCTGATGGACCAGTTATGACCAACTCTCTTACCGCTGCTGATTGGTGCCGCTCTAAGgtaacactatttttgtctctgaaccaactctttaaatttaaatttcagAAGACTTACCCAAACGATAAGACTATCATCTCTACTTTCAAGTGGTCTTACACTACTGGAAACGGAAAGCGCTACCGCTCTACCGCTCGCACTACCTACACCTTCGCTAAGCCAATGGCCGCCAACTACCTCAAGAACCAACCAATGTACGTCTTCCGTAAGACCGAGCTCAAGCACTCCAAGACCGAGCTCAACTTCAAGGAGTGGCAAAAGGCCTTCACCGACGTCATGGGAATGGACGAGCTCTACAAGTAAaattcactcgtgattcattgcccaattgataatt

The encoded fusion protein comprises the entire CED-3 ORF (ending with PEARNSAV) followed by a flexible linker sequence (GSGAGASGG) and then the mNG sequence

(beginning with MPSKGEE and ending with MDELYK*).

***ced-4(dx226)***

Cas9 crRNA seqeunce UUGCAUGCUGUUAAAACCUC was used to target cleavage near the 3' end of *ced-4* coding sequence.

The repair template was as folllows, with homolog arms underined:

aattctatgactccctcaaaaattttgcatgctgtggttctggagccggggcttcaggcggtATGCCAGGATCTAAGGGAGAAGAGGATAACATGGCTTCCCTTCCAGCTACTCACGAACTTCATATTTTCGGATCTATCAACGGAGTTGATTTCGATATGGTTGGACAGgtaagtttaaacatatatatactaactaaccctgattatttaaattttcagGGAACTGGAAATCCAAACGATGGATACGAAGAGCTTAACCTTAAGTCTACCAAGGGAGATCTTCAATTCTCTCCATGGATCCTTGTCCCACACATCGGATACGGATTCCACCAATACCTCCCATACCCAGACGGAATGTCCCCATTCCAAGCTGCTATGGTTGATGGATCTGGATACCAAGTTCACCGCACTATGCAATTCGAGGATGGAGCTTCCCTTACCGTTAACTACCGCTACACTTACGAAGGATCTCACATCAAGGGAGAAGCTCAAGTTAAGGGAACTGGATTCCCAGCTGATGGACCAGTTATGACCAACTCTCTTACCGCTGCTGATTGGTGCCGCTCTAAGgtaacactatttttgtctctgaaccaactctttaaatttaaatttcagAAGACTTACCCAAACGATAAGACTATCATCTCTACTTTCAAGTGGTCTTACACTACTGGAAACGGAAAGCGCTACCGCTCTACCGCTCGCACTACCTACACCTTCGCTAAGCCAATGGCCGCCAACTACCTCAAGAACCAACCAATGTACGTCTTCCGTAAGACCGAGCTCAAGCACTCCAAGACCGAGCTCAACTTCAAGGAGTGGCAAAAGGCCTTCACCGACGTCATGGGAATGGACGAGCTCTACAAGtaaaacctcaggcaatattgtacacgattatattcccctcg

The encoded fusion protein comprises the entire CED-4 ORF (ending with KNFACC) followed by the flexible linker sequence (GSGAGASGG) and then mNG

(MPSKGEE...MDELYK*).

***ced-9(dx236)***

The Cas9 crRNA AUUUUAGAUGACACGCUGCA was used to target cleavage near the 5' end of *ced-9* coding sequence.

The repair template was as folllows, with homology arms underined:

gtatattatgattatgaaaacgaataaaaattttagATGCCAGGATCTAAGGGAGAAGAGGATAACATGGCTTCCCTTCCAGCTACTCACGAACTTCATATTTTCGGATCTATCAACGGAGTTGATTTCGATATGGTTGGACAGgtaagtttaaacatatatatactaactaaccctgattatttaaattttcagGGAACTGGAAATCCAAACGATGGATACGAAGAGCTTAACCTTAAGTCTACCAAGGGAGATCTTCAATTCTCTCCATGGATCCTTGTCCCACACATCGGATACGGATTCCACCAATACCTCCCATACCCAGACGGAATGTCCCCATTCCAAGCTGCTATGGTTGATGGATCTGGATACCAAGTTCACCGCACTATGCAATTCGAGGATGGAGCTTCCCTTACCGTTAACTACCGCTACACTTACGAAGGATCTCACATCAAGGGAGAAGCTCAAGTTAAGGGAACTGGATTCCCAGCTGATGGACCAGTTATGACCAACTCTCTTACCGCTGCTGATTGGTGCCGCTCTAAGgtaacactatttttgtctctgaaccaactctttaaatttaaatttcagAAGACTTACCCAAACGATAAGACTATCATCTCTACTTTCAAGTGGTCTTACACTACTGGAAACGGAAAGCGCTACCGCTCTACCGCTCGCACTACCTACACCTTCGCTAAGCCAATGGCCGCCAACTACCTCAAGAACCAACCAATGTACGTCTTCCGTAAGACCGAGCTCAAGCACTCCAAGACCGAGCTCAACTTCAAGGAGTGGCAAAAGGCCTTCACCGACGTCATGGGAATGGACGAGCTCTACAAGGGATCAGGCgccggtgcttcagccggatctggcgggtctggagctatgacGcgTtgcacggcggacaactcgctgacgaatccggcgta

The tagged protein begins with mNG (MPGSKGEED...MDELYK), followed by a flexible linker (GSGAGASAGSGGSGA) and then full-length CED-9 MTRCTADNS… .

**Generation of deletion alleles of *ced-3* and *ced-9***

*ced-3(dx211)* and *ced-3(dx213)* were generated by injection of a mix containing a) Cas9 b) *ced-3* crRNA (UCAAAUCGUACUCUGACUAC)+ tracrRNA (5 micromolar) c) *ced-3* crRNA (GCUGACUGAAGUCAAUAAGA) + tracrRNA (5 micromolar) d) oligo repair template, (tttcagactaaatcgaaaatcaaatcgtactctgacAGGTCGCTTGTGGATTTCAGACATCACAGGGATCG; 10 micromolar) and e) *unc-29* crRNA (seq) + tracrRNA (10 micromolar). In the case of *ced-3(dx211)*, the mix also contained crRNA + tracRNA for deletion of *ced-9* to generate *ced-9(dx210)* (aUUUUagaUgacacgcUgca and UcgUUggagUcgUcgUgUgU, each at 5 micromolar), plus repair template (attatgaaaacgaataaaaattttagatgacacgcgtgggcggatgatgttcagcttgaagtaacgtatt; 10 micromolar); furthermore, *ced-9(dx210)* was generated in a *ced-4(dx226)* background. F1 *unc-29* animals were singled and their progeny screened by PCR for deletion of *ced-3*.

Candidate deletion homozygotes were sequenced to assess the deletion junction. The sequence of *ced-3(dx213)* exactly matches the repair template, thus corresponding to a deletion of 4300 bp that begins 46 bp before the ATG and ends 44 bp before the end of exon 7. This removes 93% of coding sequences and is expected to be null mutation. The sequence of *ced-3(dx211)* deletes an additional 26 bp at this location (deleted nucleotides underlined: tttcagactaaatcgaaaatcaaatcgtactctgacAGGTCGCTTGTGGATTTCAGACATCACAGGGATCG.

*ced-9(dx215)* was generated using essentially the same procedure as above, except that *ced-3*-specific reagents were not included in the mix and this was done in a *ced-4(n1162)* background. *ced-9(dx210)* and *ced-9(dx215)* were confirmed by sequencing to match the repair template, corresponding to a 1873 bp deletion that removes 96% of coding sequence and thus are expected to be null alleles.

**Supplemental figure**

**Figure S1**

CED-3

Atp-1

**Figure S1.** Comparison of predicted mitochondrial targeting signals in CED-3 and Atp1p. Internal mitochondrial targeting sequences were identified using the online tool described by Jung et al. (Jung et al. 2024), available at https://csb-imlp.bio.rptu.de/. CED-3 sequence is CED-3a from WormBase (WS294) and Atp1p is CAA84924.1 ATP1 [Saccharomyces cerevisiae]. Atp1p is known to localize to the outer mitochondrial membrane (Backes et al. 2018).
